# Differences between racing and non-racing drivers: A simulator study using eye-tracking

**DOI:** 10.1371/journal.pone.0186871

**Published:** 2017-11-09

**Authors:** Peter M. van Leeuwen, Stefan de Groot, Riender Happee, Joost C. F. de Winter

**Affiliations:** Delft University of Technology, Department of Biomechanical Engineering, Faculty of Mechanical, Maritime and Materials Engineering, Mekelweg 2, CD Delft, The Netherlands; Beihang University, CHINA

## Abstract

Motorsport has developed into a professional international competition. However, limited research is available on the perceptual and cognitive skills of racing drivers. By means of a racing simulator, we compared the driving performance of seven racing drivers with ten non-racing drivers. Participants were tasked to drive the fastest possible lap time. Additionally, both groups completed a choice reaction time task and a tracking task. Results from the simulator showed faster lap times, higher steering activity, and a more optimal racing line for the racing drivers than for the non-racing drivers. The non-racing drivers’ gaze behavior corresponded to the tangent point model, whereas racing drivers showed a more variable gaze behavior combined with larger head rotations while cornering. Results from the choice reaction time task and tracking task showed no statistically significant difference between the two groups. Our results are consistent with the current consensus in sports sciences in that task-specific differences exist between experts and novices while there are no major differences in general cognitive and motor abilities.

## Introduction

Motorsport has evolved from the recreational level into a high-profile international sport attracting millions of viewers worldwide [1,2]. The goal of a racing driver is typically to achieve the fastest possible lap time by driving the vehicle at the limit of tire grip in an optimal manner [3,4]. Unlike the extensive body of knowledge on the technological aspects of racecars, comparatively little is known about the motor, perceptual, and cognitive skills of athlete performance in motorsports [5–7]. Knowledge of these skills may aid in designing training methods for racing drivers and improve driver-vehicle interfaces for not only motorsport applications but also road vehicles (e.g., [8–10]).

### Differences between experts and non-experts

It is well established that practice is a prerequisite for achieving high levels of performance at a task. Ericsson et al. [11] reported that the amount of accumulated practice for expert musicians exceeded 10,000 hours at the age of 20 years. Similar findings in other domains [12] have resulted in the notion that 10,000 hours of deliberate practice is required to obtain expert-level performance. However, a recent meta-analysis by Macnamara et al. [13] showed that deliberate practice accounts for only 18% of the variance in sports performance. These authors recommended that in order to understand the determinants of expertise, findings from cognitive ability, personality psychology, behavioral genetics, and sports sciences need to be considered.

The literature suggests that experts do not differ from non-experts in elementary abilities such as visual acuity, color vision, or peripheral response time [14]. Instead, differences have been found to occur in the sport-specific processing of information [15]. A meta-analysis by Mann et al. [16] showed that experts in sports respond faster and more accurately to task-specific cues than non-experts (see also [17]). For example, experts in interceptive sports (e.g., tennis, soccer) are well able to predict the future behavior of the ball based on the opponent’s movements [18–21].

### Relevance of perceptual-cognitive skills in (high-speed) driving

A large amount of research has been dedicated to studying the effects of visual stimuli on steering control. Land and Lee [22] formulated a model that describes how regular (i.e., non-racing) drivers steer their vehicle. These authors found that drivers directed their gaze predominantly at the tangent point (defined as the point where the inside road edge reverses direction), and they illustrated a geometrical relation between the tangent point location, corner curvature, and required steering input. The tangent point at a specific moment in time coincides with the apex point of a corner. The apex point is a fixed location on the inside road edge of a corner and is strongly related to the racing line the driver takes while cornering [23]. Other models suggest that drivers control steering by means of optical flow information [24] and that they direct their gaze on their future path, approximately 1 to 2 seconds ahead of the vehicle [25]. These results correspond to various other findings showing that humans use optical flow to perceive their heading [26,27].

Research has also shown that experienced drivers direct their gaze further ahead than novices [28,29]. Furthermore, experienced drivers exhibit greater gaze variance than novices, which can be explained by the fact that they scan the environment more actively [30,31]. Finally, it has been found that experienced drivers rely less on foveal vision and more on peripheral vision for their steering control [32].

### Previous research on racing drivers

Backman et al. [33] compared nine open-wheel racing drivers to nine rally drivers and nine regular drivers. Their results indicated differences in grip strength as measured with a dynamometer, with higher grip strengths for the rally drivers and open-wheel racing drivers compared to the normal drivers. Backman et al. [33] also found higher relative neck strength for the open-wheel racing drivers compared to the other two groups. Baur et al. [34] observed significantly faster reaction times for eight racing drivers on a reaction time task when compared to ten normal drivers. However, no significant differences were found for postural stability, leg extensor strength, arm strength, or arm endurance.

Bernardi et al. [35,36] compared the brain activity of experienced racing drivers with that of normal drivers. Eleven professional drivers and eleven age-matched drivers watched video clips from the drivers’ viewpoint of Formula One cars in an MRI scanner. Results indicated that compared to the non-racing drivers, the racing drivers showed more consistent recruitment of brain areas devoted to motor control and spatial navigation. The authors indicated that “exceptional driving abilities may acquire the acquisition of a specific behavioral and functional motor repertoire that is different from the one associated with common ‘every day driving’” (p. 9).

In a literature review on brain imaging in relation to driving expertise, Lappi [10] argued that differences in brain activity between racing drivers and regular drivers may be due to the racing drivers’ task familiarity. The author argued that “whereas for a naïve participant steering a series of bends may effectively be reduced to a simple path-following visuomotor routine, to the expert with detailed survey knowledge of the track and a deep understanding of cornering techniques (cued by landmarks), many additional cognitive operations may be performed”. The regions of brain activity indicated by Bernardi et al. [35] are used during the control of pursuit and saccadic eye movements [10], suggesting that differences in brain activity are related to eye movement strategies.

In an on-track study, Land and Tatler [37] measured the eye movements of a professional single-seater racing driver while driving at racing speed. They found that the driver directed his gaze at a horizontal offset from the tangent point and that this offset was different for each corner. These findings illustrate that the tangent point itself was not the main area of visual attention while driving through corners. The authors also found a strong correlation between the driver’s head rotation (in yaw) and the vehicle’s rotational speed approximately one second later, and that the eyes-in-head angle remained relatively constant throughout the lap. They further argued that the driver used this relationship between the vehicle’s rotational speed and the visual information to control the vehicle path.

### Aim of this study

Previous findings in the domain of racing drivers’ expertise have mainly focused on physiological differences between racing drivers and normal drivers. Furthermore, one study has reported the eye movement behavior of a single racing driver. No studies seem to exist on the task-specific driving skills and the processing of visual information of racing drivers compared to normal drivers.

In our racing simulator experiment, we investigated differences in car control, visual strategy, and driving performance between racing drivers and non-racing drivers who completed four sessions on a racing circuit. To evaluate both groups on non-domain specific motor skills, we tested their performance on a first-order dynamic tracking task and a choice reaction time task.

We expected the racing drivers to show better driving performance in terms of faster lap times and fewer crashes. Additionally, we expected the racing drivers to adapt their path strategy to specific sections of the circuit, aiming to benefit from the racing line. Moreover, based on Land and Tatler [37], we anticipated that the racing drivers would direct their gaze less often at the tangent point as compared to the normal drivers. Finally, due to task familiarity, we expected the racing drivers to experience lower self-reported workload than the normal drivers.

## Materials and methods

### Participants

Seven male racecar drivers competing in international race categories (Formula 3, GP2, and Porsche Supercup) and ten males from the Delft University of Technology campus were recruited for this experiment. Before starting the experiment, participants completed an intake questionnaire consisting of general items (e.g., age, wearing contacts or glasses, simulator experience, racing games experience), 14 items detailing their racing experience (e.g., number of years racing in cars, number of participated go-kart races), 8 items about their driving experience (e.g., annual mileage, number of accidents, number of traffic fines), and 11 items about violations and errors. The 11 items were derived from the Driving Habits Questionnaire [38] and the Driving Behaviour Questionnaire [39].

The racing drivers’ mean age was 19.9 (*SD* = 1.8), and the non-racing drivers’ mean age was 21.6 (*SD* = 1.7) years. Fifteen participants (six racing drivers and nine non-racing drivers) were in possession of a driver’s license. Participants had an average annual mileage of 14,070 km (*SD* = 19,196). The racing drivers had on average 8.4 (*SD* = 3.0) years of experience racing in cars and go-karts, and on average had participated in 82.3 (*SD* = 64.5) go-kart races during their go-karting career. Both the racing drivers and non-racing drivers reported playing racing games, on average for 3.3 (*SD* = 3.2) and 0.2 (*SD* = 0.6) hours per week, respectively. All racing drivers and one non-racing driver reported previous experience in racing simulators. All participants provided informed written consent before starting the experiment, and the research was approved by the Human Research Ethics Committee of the Delft University of Technology.

### Apparatus

The experiment was conducted in a fixed-base racing simulator based on a Tatuus Formula Renault 2.0 chassis used for racecar driver training (SimDelft, the Netherlands). The simulator was equipped with the components originating from the original car: steering wheel, throttle pedal, brake pedal, custom-made seat. The steering wheel force feedback was provided by a control loader (SimSteering v1), and the brake and throttle pedal passive stiffness resembled those of the original car. The simulator was equipped with a dashboard on the steering wheel showing speed, engine rpm, lap, and lap sector times. The visual system consisted of three 55-inch Plasma screens (Panasonic TX-P55VT30E, refresh rate of 60 Hz) spanning a horizontal and vertical field of view of 130 and 27 degrees, respectively. Each screen had a resolution of 1920 x 1080 pixels, and the simulation ran with a frame rate exceeding 100 frames per second. Auditory feedback was provided through headphones. The virtual environment, vehicle dynamics, and force feedback were simulated by rFactor software (v1.255). We used the rTrainer vehicle model, a rear wheel driven formula-style racecar (115 bhp, 573 kg). All driving aids (ABS, ESC, traction control) were disabled, and gear shifting was automated.

**Fig 1:**
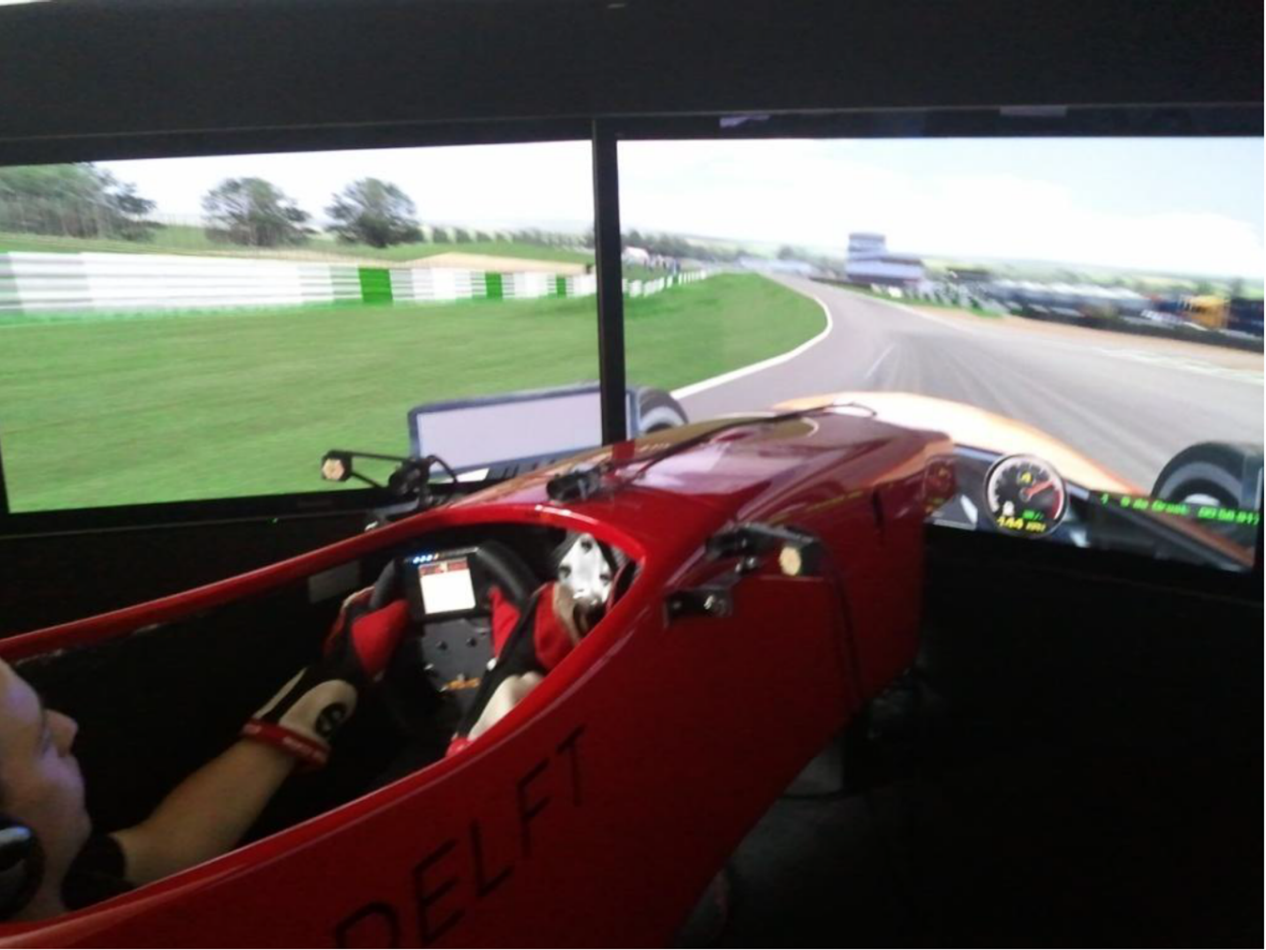
Overview of the racing simulator and eye tracker.

Head and eye movements were recorded using a remote eye tracker from Smart Eye (v5.8), consisting of three remote mounted cameras (Sony XC-HR50) and two infrared illuminators mounted above the steering wheel on the simulator chassis (see Fig 1 (left) for a photo of the racecar simulator and eye tracker). Eye-tracker data were sampled at 60 Hz, and all eye-tracker and simulator data were recorded and stored synchronously at 100 Hz.

### Procedures

Before the simulator experiment commenced, participants received a paper handout explaining the experiment and procedures, and filled out the intake questionnaire. After completing the intake questionnaire, participants were seated in front of a desktop computer, received the instructions for the Deary-Liewald choice reaction time (CRT) task [40], and completed 10 practice and 40 measurement trials. After completion of the CRT task participants received the instructions for the visual-motor task (MMSLab) and performed six trials of the MMSLab task [41].

After the MMSLab task, participants entered the simulator cockpit and received oral instructions regarding the simulator operation, the procedure to resume driving after a road departure, and the steering wheel dashboard information (e.g., vehicle speed, current lap time, best lap time). A series of head and eye movements followed to calibrate the eye-tracker equipment before the first session commenced. Participants drove four sessions, each session lasting 10 minutes. After the first and the third session, a 5-minute break followed during which participants remained seated in the simulator and completed the NASA-TLX questionnaire [42] for measuring workload. After the second session, a 10-minute break followed during which participants completed the NASA-TLX questionnaire outside of the simulator. After completing the NASA-TLX questionnaire of the final session, participants completed a questionnaire concerning their subjective performance and the perceived vehicle handling quality for each corner.

### Experimental tasks

The choice reaction time task consisted of four horizontally placed white squares displayed on a PC-monitor and a keyboard with four assigned keys, one key corresponding to each square’s location. Participants were requested to press the corresponding key as quickly as possible after one of the four squares showed a black cross. The MMSLab task consisted of a one-dimensional (horizontal) compensatory tracking task. A green error symbol and a white target box were displayed on a black background. Participants were required to use the computer mouse to minimize the error (distance between the error symbol and the static target box). The controlled dynamics of the mouse were a first-order integrator, whereby the object’s velocity was proportional to mouse displacement.

During the simulator sessions, participants were instructed to drive the fastest possible lap time while keeping their vehicle on the road (i.e., they were not allowed to cut corners). Participants were instructed to use the steering wheel, throttle pedal, and brake pedal to operate the vehicle, and they were informed that gear shifting was automated. The sessions started with the vehicle from a standstill in the pit lane (participants were required to drive the vehicle onto the track after the session started).

### Driving environment

**Fig 2:**
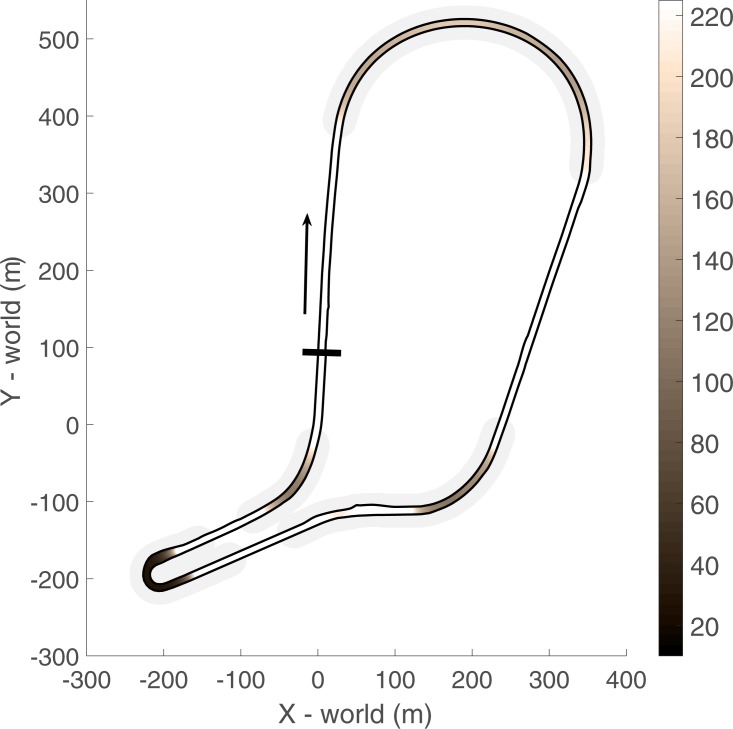
Overview of the circuit layout and centerline corner radius in meters. The start/finish line and driving direction are indicated by the black line and arrow, respectively. Grey shaded areas indicate the corner segments.

All driving sessions took place on a virtual representation of the racetrack Mallory Park in Leicestershire, United Kingdom, as in Land and Tatler [37]. None of the participants had driven on this racetrack before. The circuit, with a length of 2,172 m, consisted of a large-radius right-hand corner (minimum corner radius = 128 m), a right corner, directly followed by a short left corner (minimum corner radius = 120 m), a sharp hairpin (minimum corner radius = 14 m), and a left-hand corner (minimum corner radius = 118 m). See Fig 2 for an overview of the circuit layout and the corner radii for the various corners.

### Dependent measures

Prior to the analysis, data from the steering sensor, brake pedal, and throttle pedal were low-pass filtered at a 3 Hz cutoff frequency to remove high-frequency noise. Furthermore, eye and head movements were low-pass filtered at 10 Hz and 5 Hz cutoff frequencies, respectively. Missing eye-tracker data (e.g., due to camera obstruction from hands on the steering wheel, or eye blinks) were removed from the dataset [43]. Gaze data from 0.2 s before to 0.2 s after segments of missing data were also removed. If the eye-tracker data loss of a session exceeded 60%, the complete eye-tracker dataset of the respective session was removed from the analysis. The following dependent measures were calculated from the fastest lap data for each participant and session:

#### Reaction time and visual-motor performance

Choice reaction time (CRT; ms) and root mean square of the MMSLab tracking error (-) were calculated as measures of basic cognitive skill and visual-motor performance. Reaction time is related to general cognitive ability [40] and has frequently been used as a performance measure of motor reaction tasks [33,36]. The root mean squared MMSLab tracking error was calculated as the root of the arithmetic mean of the squares of each measured tracking error.

#### Driving performance

Drivers were assessed on performance measures of best lap time (s), median lap time (s) (m/s), and the number of road departures. A road departure was counted as an event in which all four wheels were outside of the road boundaries. If a road departure occurred, the lap was declared invalid, and the data were excluded from the analysis. A maximum of one road departure per lap was counted.

#### Vehicle control

The mean steering speed (deg/s) and throttle variance (%^2^) were used as measures of control activity and consistency when driving in corners [44,45]. Furthermore, the maximum brake pedal position on a scale of 0 (minimum) to 100% (maximum) was used as a measure of braking performance [46]. The time from the initial brake pedal actuation to the maximum brake pedal position during a braking maneuver was used as a measure of brake efficiency [9].

#### Eye and head movements

Gaze direction and head rotation data were collected to compute the difference between the horizontal gaze angle and the angle of the line from the vehicle center to the tangent point [47]. A positive value means that drivers looked at the right of the tangent point, and a negative value means that drivers look to the left of the tangent point. The tangent point locations were calculated from the circuit edge geometry and the center of gravity position of the vehicle. As mentioned above, the tangent point bears a close relationship to the apex point, used by racing drivers as a visual reference point while driving [37].

#### Subjective measures

The NASA-TLX (0–100) questionnaire [42] was used to assess the participants’ self-reported workload on the following six items: mental demand, physical demand, temporal demand, performance, effort, and frustration. The responses were marked on a 21-tick horizontal bar with anchors on the left (*very low*) and right sides (*very high*). For the performance item, the anchors (*perfect*) and (*failure*) on the left and right side were used. After the simulator sessions, participants were requested to rate the handling quality of the vehicle for each individual corner by answering the following question: “The vehicle handling was good” on the following levels: disagree, somewhat disagree, neutral, somewhat agree, agree.

### Statistical analysis

Means and standard deviations across participants were computed for the best lap of each participant in each session. Differences between sessions were statistically analyzed with paired *t* tests (α = 0.05). The results of the number of road departures and the averaged absolute vehicle yaw rate during road departures were fractionally ranked [48] per session, because of the skewed distribution of these variables.

## Results

The eye-tracker data for 1 of out 17 participants were removed due to malfunctioning of the eye tracker. Furthermore, four sessions of eye-tracker data were removed due to the data loss exceeding the 60% threshold. Of the remaining 60 sessions (i.e., 17 participants x 4 sessions– 8 missing sessions), 13% of eye-tracker data were removed from the analysis (e.g., due to blinks). Data from the CRT task of one participant (from the racing drivers group) were removed due to failure to adhere to the task instructions (i.e., the participant misunderstood the task instructions). Furthermore, the MMSlab data from one participant (from the racing drivers group) were unavailable due to a data logging error.

### Reaction time and visual-motor performance

No statistically significant differences were found in the CRT between the racing drivers and the non-racing drivers. The average reaction time (averaged across 40 trials) was 431.6 ms (*SD* = 35.8 ms) for the racing drivers and 439.5 ms (*SD* = 34.2 ms) for the non-racing drivers (*t*(14) = 0.437, *p =* 0.6689). Furthermore, the racing drivers’ results of the motor skill task did not significantly differ from the non-racing drivers. Specifically, the RMS error (averaged across all six trials) was 0.222 (*SD* = 0.078) for the racing drivers and 0.178 (*SD* = 0.033) for the non-racing drivers (*t*(14) = 1.588, *p =* 0.1346).

### Driving performance

**Fig 3:**
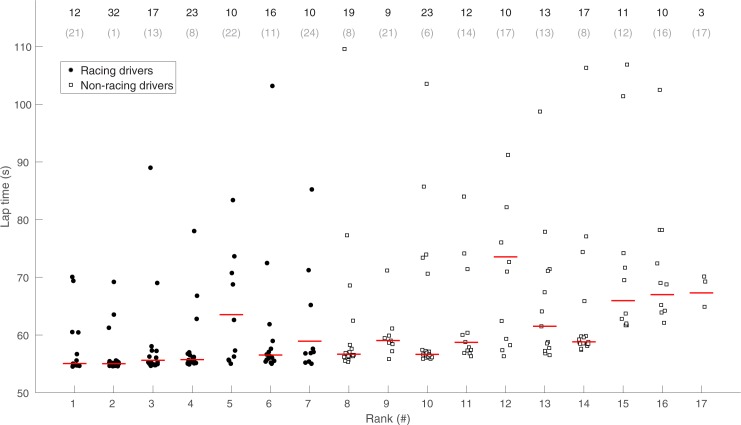
Overview of all lap times of all sessions per participant. The ranking is based on the best lap time, and each rank corresponds to one participant. The lap time median per participant is shown in red. Markers were given a random offset from -0.25 to 0.25 on their rank, to reduce the overlap of markers. The numbers above the graph correspond to the total number of completed laps and the number of discarded laps (in parentheses) per participant.

The racing drivers drove statistically significantly lower best lap times than the non-racing drivers (Table 1). In Fig 3 all included lap times for all participants are shown, ranked per fastest lap of the participant. The figure also shows the number of laps excluded for each participant; this number provides an indication of individual differences in the number of road departures.

**Table 1:** Session means (standard deviations in parentheses) of the dependent measures for the four sessions. *p* values are indicated for comparisons between the both groups of drivers. Pearson correlation coefficients are shown between the first and fourth session and the third and fourth session.

Dependent measures						Pearson correlation
		Session 1	Session 2	Session 3	Session 4	S3-S4
Best lap time (s)						
	Racing drivers	55.9 (0.85)	55.3 (0.35)	54.9 (0.28)	54.8 (0.21)	0.714
	Non-racing drivers	57.9 (0.67)	59.0 (2.78)	59.1 (3.95)	58.1 (2.55)	0.899
	*p* value	**0.0007**	**0.0033**	**0.0149**	**0.0047**	
Median lap time (s)						
	Racing drivers	56.5 (0.68)	55.6 (0.29)	55.2 (0.26)	55.2 (0.27)	0.071
	Non-racing drivers	58.5 (0.95)	61.2 (4.39)	59.9 (3.83)	59.2 (3.26)	0.942
	*p* value	**0.0009**	**0.0047**	**0.0059**	**0.0055**	
Road departures (#)*						
	Racing drivers	4.86 (2.19)	5.29 (3.04)	4.00 (2.71)	4.14 (2.41)	0.111
	Non-racing drivers	5.90 (0.57)	3.90 (1.79)	3.80 (2.10)	3.60 (1.58)	0.578
	*p* value	0.6118	0.3392	0.9252	0.5374	
Steer speed corners (deg/s)						
	Racing drivers	20.9 (7.27)	18.4 (5.66)	16.5 (4.21)	16.8 (4.00)	0.752
	Non-racing drivers	17.8 (4.33)	13.7 (2.90)	12.4 (3.39)	12.0 (3.44)	0.933
	*p* value	0.3840	**0.0404**	**0.0432**	**0.0187**	
Throttle variance corners (%^2^)						
	Racing drivers	1618 (117)	1699 (46.0)	1664 (100)	1719 (115)	0.571
	Non-racing drivers	1421 (114)	1357 (137)	1343 (140)	1366 (159)	0.788
	*p* value	**0.0108**	**<0.0001**	**0.0001**	**0.0002**	
Max brake (0–100)						
	Racing drivers	74.6 (14.8)	78.2 (15.2)	82.9 (14.7)	83.0 (14.1)	0.964
	Non-racing drivers	79.4 (17.5)	65.8 (16.6)	75.2 (13.6)	69.4 (16.5)	0.835
	*p* value	0.6062	0.1382	0.2873	0.0960	
Mean absolute head yaw (deg)						
	Racing drivers	7.64 (4.12)	8.27 (3.16)	7.81 (3.84)	11.17 (4.35)	0.800
	Non-racing drivers	4.20 (2.13)	5.16 (2.57)	4.03 (2.11)	4.60 (2.49)	0.950
	*p* value	0.1204	0.0555	**0.0279**	**0.0025**	
TLX mental demand (0–100)						
	Racing drivers	39.3 (25.2)	41.7 (28.9)	41.7 (24.6)	40.0 (21.7)	0.993
	Non-racing drivers	56.5 (15.8)	56.5 (18.9)	56.0 (24.0)	64.0 (18.4)	0.651
	*p* value	0.1031	0.2316	0.2713	**0.0328**	
TLX physical demand (0–100)						
	Racing drivers	8.6 (10.7)	12.9 (12.9)	11.4 (9.9)	16.4 (12.5)	0.881
	Non-racing drivers	41.5 (22.2)	55.0 (22.2)	58.0 (21.5)	61.5 (24.0)	0.909
	*p* value	**0.0026**	**0.0004**	**<0.0001**	**0.0004**	
TLX temporal demand (0–100)						
	Racing drivers	19.3 (16.9)	16.4 (17.7)	20.7 (20.3)	25.0 (16.8)	0.817
	Non-racing drivers	51.0 (14.1)	51.0 (18.1)	50.5 (19.4)	60.0 (19.2)	0.959
	*p* value	**0.0008**	**0.0014**	**0.0079**	**0.0015**	
TLX performance (0–100)						
	Racing drivers	47.1 (19.6)	47.1 (18.7)	31.4 (18.4)	34.3 (21.5)	0.434
	Non-racing drivers	68.0 (21.4)	52.0 (16.9)	54.0 (15.6)	45.5 (13.4)	0.798
	*p* value	0.0584	0.5840	**0.0155**	0.2035	
TLX effort (0–100)						
	Racing drivers	41.4 (30.1)	53.6 (23.9)	51.4 (21.0)	59.3 (16.7)	0.800
	Non-racing drivers	63.5 (11.3)	68.0 (13.2)	72.0 (10.3)	74.5 (12.1)	0.808
	*p* value	**0.0495**	0.1295	**0.0166**	**0.0451**	
TLX frustration (0–100)						
	Racing drivers	28.6 (32.2)	39.3 (30.3)	29.3 (24.2)	30.7 (25.9)	0.703
	Non-racing drivers	56.5 (23.7)	45.0 (26.7)	47.0 (27.5)	41.5 (16.7)	0.750
	*p* value	0.0566	0.6867	0.1910	0.3106	

*Note: For the road departures the Spearman correlation was computed.

The session means, standard deviations, and Pearson correlation coefficients between the third and the fourth session are shown in Table 1 for both the racing drivers and the non-racing drivers. Similar to Fig 2, the table shows significant differences in best and median lap times of the racing drivers compared to the non-racing drivers in all four sessions. Furthermore, both the racing drivers (*t*(6) = 3.55, *p =* 0.012) and the non-racing drivers (*t*(9) = 2.80, *p =* 0.021) significantly improved their best lap time from the first to the last session. The Pearson correlations in Table 1 indicate that between-subject differences remain consistent over the third and the last session.

There was no significant difference in the number of road departures between the racing drivers and the non-racing drivers. On average, the racing drivers and the non-racing drivers had approximately 4.6 and 4.3 road departures per session, respectively. During the road departures, the averaged absolute vehicle yaw rate was significantly higher (*t*(15) = 4.95, *p <* 0.0002) for the non-racing drivers (272 deg/s, *SD* = 172 deg/s) compared to the racing drivers (124 deg/s, *SD* = 67 deg/s). A higher vehicle yaw rate during a road departure indicates a loss of control.

The racing drivers showed higher steering speeds and higher throttle variance while cornering when compared to the non-racing drivers, reaching statistical significance in all sessions except the first session for the steering speeds. No significant difference was found in the maximum brake position.

The subjective measures indicated a statistically significantly lower physical and lower temporal demand for the racing drivers as compared to the non-racing drivers. The mental demand, performance, and effort were also lower for the racing drivers than for the non-racing drivers, with each item reaching significance in at least one of the four sessions. The non-racing drivers reported significantly (*t*(15) = 4.792, *p* = 0.0002) better handling qualities of the vehicle, scoring the vehicle handling (from 0 to 5) on average with 3.8 (*SD* = 0.58) compared to 2.6 (*SD* = 0.37) for the racing drivers.

**Fig 4:**
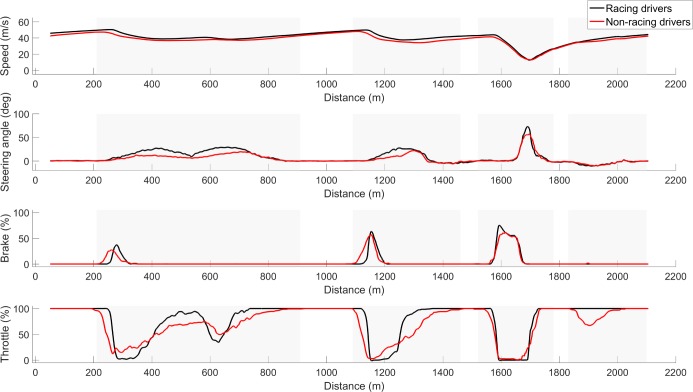
From top to bottom, overview of vehicle speed, steering angle, brake position, and throttle position as a function of traveled distance for the racing drivers and non-racing drivers averaged across both groups for the fastest laps of each of the four sessions. Grey shaded areas indicate the four corners.

Fig 4 shows the vehicle speed, steering wheel angle, brake position, and throttle position, respectively as a function of traveled distance in the lap. On average, the racing drivers’ vehicle speed in the corners was higher than for the non-racing drivers’. The figure also shows higher steering wheel angles for the racing drivers compared to the non-racing drivers. Moreover, in the first two corners, the racing drivers on average pressed the brake at a later position than the non-racing drivers did. Finally, the throttle position shows that racing drivers more often drove full (100%) throttle. It can also be seen that the racing drivers drove the fourth corner at 100% throttle, whereas some non-racing drivers released the throttle in this corner.

### Vehicle control

**Fig 5:**
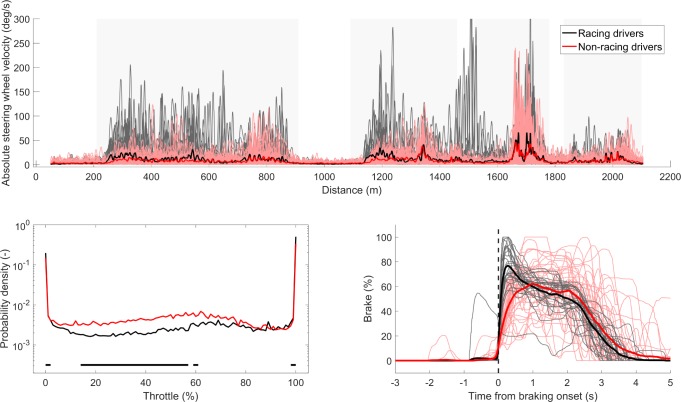
Top: absolute steering wheel velocity as a function of traveled distance of the fastest lap of each session (*N* = 28 for the racing drivers, *N* = 40 for the non-racing drivers). Individual lines are shown as well as the group means with a thick line type. Grey shaded areas indicate the four corners. Lower left: Probability density of the throttle position averaged across the fastest laps of each session, for the racing drivers and non-racing drivers. Significant differences (*p* < 0.01) are indicated by the black horizontal line at the bottom of the figure. Lower right: Brake position traces for the fastest lap of each session, for the racing drivers and non-racing drivers. A temporal shift was applied to the onset of braking (defined as brake position > 10). Individual participants’ brake positions are shown, as well as the group means indicated by the thicker line. The vertical dashed line indicates the brake onset time, at t = 0 s.

The difference in vehicle control between the racing drivers and non-racing drivers is illustrated in Fig 5. It can be seen that the racing drivers drove the vehicle with higher steering speeds in all corners. Furthermore, the racing drivers drove a smaller amount of time with throttle positions between 20% and 60% than the non-racing drivers. This can be explained by a difference in throttle style: Compared to the non-racing drivers, the racing drivers held the throttle position longer at 100% and released to 0% at a later position in the corners (see Fig 4).

Fig 5 also shows the brake pedal position for the third corner, illustrating the differences in brake pedal position build-up, modulation, and consistency between the racing drivers and the non-racing drivers. The racing drivers reached the maximum brake position after 0.38 s (*SD* = 0.15 s), which is significantly faster (*t*(15) = 6.71, *p <* 0.001) than the non-racing drivers, who reached the maximum brake pedal position after 1.12 s (*SD* = 0.26 s). Furthermore, the racing drivers showed a more distinct peak in the brake pedal position, whereas the non-racing drivers showed more variability between the participants.

**Fig 6:**
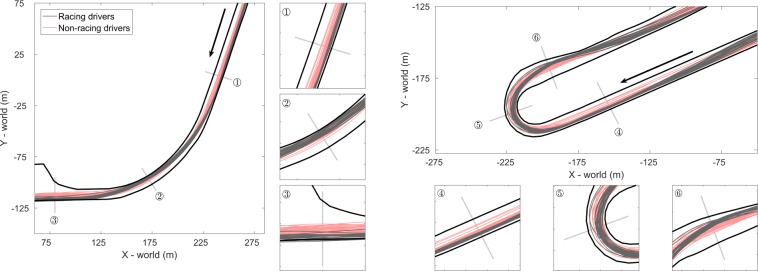
Individual paths of the vehicle center of the racing drivers (N = 28) and non-racing drivers (N = 40) for the fastest laps of each session for the second (left) and third (right) corner, the driving direction is indicated by the black arrow. The three panels indicate the start (1), middle (2), and the end of the corner (3) and corresponds to an area of 40 by 40 m.

The vehicle paths for the racing drivers and the non-racing drivers for the second and third corner are show in Fig 6. It can be seen that compared to the non-racing drivers, the racing drivers adopted a traditional racing line in each of the corners, by approaching the corner from the outside (i.e., the left side of the circuit in case of a right-hand corner). At the middle or apex of the corner, the racing drivers drove more to the inside of the corner inside, and when exiting the corner, they consistently used the outside portion of the circuit. Compared to the racing drivers, the non-racing drivers adhered more to the centerline of the road and adopted a racing line to a lesser extent.

### Eye and head movements

**Fig 7:**
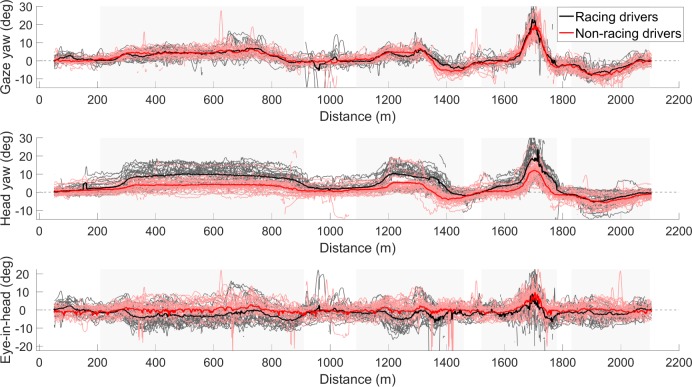
Gaze yaw angle (top), head yaw angle (middle), and eye-in-head angle (bottom) during the fastest overall lap of each session and racing drivers (*N* = 26) and non-racing drivers (*N* = 34). Individual lines are shown as well as the group means by the thick line. Positive values correspond to rotation to the right. The eye-in-head was determined as the difference between the gaze yaw angle and the head yaw angle and illustrates the orientation of the eye with respect to the head. Grey shaded areas indicate the four corners and a gray dashed line in the figures references to zero.

The individual and averaged gaze yaw angle, for both the racing drivers and the non-racing drivers, as a function of traveled distance per lap is illustrated in Fig 7. It can be seen that there were only small differences in the gaze yaw angle between the two groups. However, there were large differences in the head yaw angle for the racing drivers compared to the non-racing drivers (see also Table 1). The racing drivers turned their head nearly twice as much as the non-racing drivers while cornering.

This similar gaze yaw angle and difference in head yaw angle between the racing drivers and the non-racing drivers yields a difference in eye-to-head angle, which is illustrated in the lower pane of Fig 7. It can be seen that racing drivers showed slightly negative eye-in-head angles compared to the non-racing drivers, who predominantly have a near zero eye-to-head angle (except for the small corner radius corner at approximately 1700 m). In summary, the racing drivers steered their head more into the corners than the non-racing drivers.

**Fig 8:**
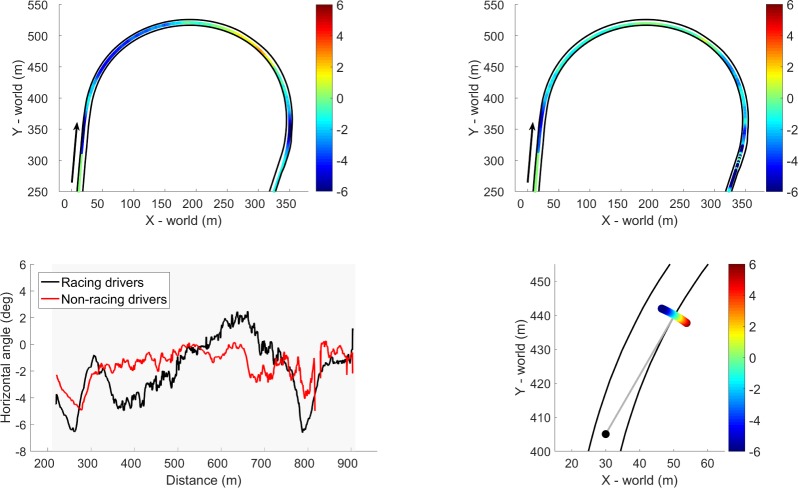
Difference between the horizontal gaze angle and the tangent point angle as a function of track position for the racing drivers (top left) and non-racing drivers (top right) averaged across all sessions and fastest laps. The black arrow indicates the driving direction. The lower left panel shows the horizontal gaze angle with respect to the tangent point, averaged across all sessions and fastest laps for both the racing drivers and the non-racing drivers. The lower right panel shows a definition of the horizontal gaze angle, the tangent point, and the color scaling.

The tangent point analysis reveals a difference in gaze between the racing drivers and the non-racing drivers in the first corner. In Fig 8, the difference in the horizontal gaze angle and the angle between the vehicle and the tangent point is shown for both the racing drivers and the non-racing drivers. The figure shows that the racing drivers varied their gaze direction as a function of traveled distance, whereas the non-racing drivers kept a more constant gaze location, close to the vicinity of the tangent point. As the racing drivers entered the corner, they directed their gaze away from the tangent point towards the outside of the corner, and as they progressed through the corner they moved their gaze towards the tangent point and beyond the tangent point. As the racing drivers exited the corner, they directed their gaze again towards the outside of the corner and subsequently looked again towards the tangent point.

## Discussion

In this paper, we studied the performance, control behavior, and visual behavior of seven young racing drivers in comparison with ten non-racing drivers when racing on a simulated circuit. We expected the racing drivers to perform better at racing-specific tasks (e.g., faster lap times) and based on Land and Tatler [37] we expected the racing drivers to direct their gaze less at the tangent point while cornering.

Our results confirmed that the racing drivers drove faster lap times than the non-racing drivers. In fact, there was not a single non-racing driver who drove a personal best lap that was faster than the best lap of any of the racing drivers; in other words, there was no overlap in the overall performance of both groups. Both the racing drivers and non-racing drivers significantly improved their lap times from the first session to the last session, a finding which is similar to the performance of learner drivers in other driving simulator experiments [44].

No significant differences were found in the non-domain specific choice reaction time task and tracking task. These results are consistent with the results of Bernardi et al. [36] who found no differences in a reaction time task and visuospatial task between 11 racing drivers and 11 age-matched controls. These results are also in line with various team and individual sports, showing that experts do not differ in basic visual skills when compared to non-experts [14].

Contrary to our expectation, the number of road departures did not differ between both groups from the second till the last session. This may be explained by both groups balancing their performance against the risk of having a road departure. The road departures of the racing drivers may be caused by leaving the circuit as a consequence of too much risk taking, without a loss of control. In case of the non-racing drivers, the road departures were more often a consequence of loss of control incidents, as indicated by the higher vehicle yaw rates due to the vehicle spinning.

Our results indicated a higher self-reported workload from the non-racing drivers as compared to the racing drivers, with the largest effects for the temporal demand and physical demand items. This latter finding may be explained by the physical effort required to operate the simulator. Compared to normal road cars, racing cars generate relatively high steering wheel and brake pedal forces [9], which pose physical demands that are comparable to physical demands experienced by normal athletes [33,49]. In our experiment, however, the steering wheel force feedback and brake pedal stiffness were reduced to ensure that all participants were able to complete the experiment, as verified by the similar maximum brake pedal position achieved by the non-racing drivers and the racing drivers.

Because of their higher cornering speeds, the racing drivers drove closer to the physical limits than the non-racing drivers. Due to the nonlinear characteristics of racing tires [50], the vehicle could become unstable when driving close to the friction limits of the vehicle. To control the vehicle at higher cornering speeds, more steering corrections may be required [3], which may explain why the racing drivers had higher steering activity than the non-racing drivers.

The racing drivers showed higher throttle variance during cornering and held the throttle at 0% and 100% for a larger fraction of time compared to the non-racing drivers. The racing drivers and the non-racing drivers reached comparable maximum brake pedal positions. However, compared to the non-racing drivers, the racing drivers achieved the maximum brake pedal position faster after the braking onset. These results can be explained from a time-optimality point of view: when the racing drivers decide to reduce their speed, they aim to achieve the maximum deceleration in a minimum amount of time by (1) swiftly releasing the throttle from 100% to 0%, by (2) pressing the brake pedal as fast as possible, and by (3) modulating the brake pedal position, to control the tires to their limit of adhesion to minimize the braking distance [51]. In the middle of the corner, the racing drivers keep 0% throttle to achieve the maximum lateral grip potential of the tires, see Milliken and Milliken [50] for more details on the friction circle. From the middle to the exit of the corners, the racing drivers pressed the throttle slightly later, but reached full throttle earlier, aiming to reach maximum longitudinal acceleration out of the corners.

The racing drivers adopted a more traditional racing line compared to the non-racing drivers, a finding which can also be explained from a time-optimality point of view. The racing line is considered the fastest path around a circuit and is specific to each section of the circuit and the dynamics of the vehicle [52]. Specifically, a racing line allows drivers to apply the brake pedal as late as possible and to maximize the acceleration potential of the vehicle coming out of the corners [23].

The racing drivers showed larger head rotations compared to the non-racing drivers while cornering. In other words, the racing drivers tended to turn their head more into the corner than did the non-racing drivers. These findings are consistent with Land and Tatler [37] who found that a racing driver’s eye-in-head rotation remained within 5 degrees of the head axis. In our experiment, participants did not wear helmets. It is possible that racing drivers on real tracks adapt to the restricted field of view when viewing through a helmet. Gordon and Prince [53] found up to a 22% reduction in horizontal field of view caused by full coverage helmets.

Our eye movement analysis showed a more variable gaze strategy for the racing drivers than for the non-racing drivers. The non-racing drivers adhered to a tangent point tracking strategy throughout the corner, whereas the racing drivers moved their gaze relative to the tangent point. These results are complementary to the results of a study on a real racetrack, which showed that a racing driver directed his gaze to the vicinity of the tangent point instead of at the tangent point [37]. Contrary to models of visual control of steering [22,54] in which reference points (e.g., the tangent point) are used to guide the steering input, our study shows that racing drivers vary their gaze while cornering, possibly to verify their path and to anticipate future control actions. Furthermore, the non-racing drivers may simply be looking at where they want to go [24], whereas the racing drivers may be directing their eyes to task-relevant information [55].

The differences between the racing drivers and the non-racing drivers can be explained using the three-level behavioral taxonomy of Michon [56]. At the strategic level, the racing drivers risked having road departures against achieving the optimal racing line and high cornering speeds, whereas the non-racing drivers’ road departures were caused more often by an involuntary loss of control. At the tactical level, the racing drivers showed a different gaze strategy from that of the non-racing drivers, adjusting their gaze as they drove through the corner. Furthermore, the racing drivers chose different driving lines and optimized their driving lines to increase their corner exit speeds. At the operational level, the racing drivers operated the throttle pedal such that their corner exit speeds were optimized. Finally, a more time-optimal braking strategy and a higher steering activity differentiated the racing drivers from the non-racing drivers on the operational level.

In summary, our results illustrate better performance of all racing drivers when compared to non-racing drivers. However, no differences were found between the two groups in a generic motor control task or in a choice reaction-time task. Compared to non-racing drivers, the racing drivers selected tactical and operational control strategies that result in better performance (e.g., time-optimal braking, corner exit speed optimization) and adopted control strategies that allow the vehicle to operate closer to the friction limits. On the strategic level, the racing drivers balance their performance against the risk of road departures, whereas the non-racing drivers experienced road departures due to loss of control. Eye tracking results showed that racing drivers vary their gaze while negotiating a corner whereas non-racing drivers adhered more closely to a tangent point tracking strategy. Finally, the racing drivers showed greater head rotations while cornering. Our findings are consistent with the current consensus regarding expertise in sports; our expert racing drivers excelled in the task-specific aspects of race car driving but performed similarly to a non-expert sample in more generic motor and response task. Furthermore, our eye movement results indicate a difference in perceptual-cognitive skills between the racing drivers and the non-racing drivers, which is in line with the literature [16].

Our experiment was performed in a fixed-based racing simulator, and our results may benefit from higher fidelity motion-based simulator. For instance, drivers are known to use perceived lateral acceleration to determine their corner speed [57] and possibly, racing drivers differ with respect to non-racing drivers in terms of sensitivity to such motion cues. Furthermore, our non-racing driver sample was recruited from a technical university, and the generalizability of our results may benefit from a larger and more representative sample.
